# Marginal Blepharitis

**Published:** 1907-09-14

**Authors:** 


### OPHTHALMOLOGY.

### MARGINAL BLEPHARITIS.

THIS condition is frequently met with: neglected in the early stages it becomes chronic and may be followed by sequelæ, which render the blepharitis worse, establishing a more or less vicious circle. It occurs in two forms—blepharitis squamosa and blepharitis ulcerosa.

*Blepharitis Squamosa.*—In this, small white or grey scales are seen on the lid margins around the cilia. On removing these with an alkaline lotion the skin underneath is found to be not ulcerated, but simply hyperæmic. The cilia, too, are easily pulled out, but the hair follicles not being involved they grow again. By some the condition is regarded as a seborrhœa.

*Blepharitis Ulcerosa.*—In this form the lid margin is covered with yellow crusts, beneath which the skin will be found ulcerated, red and swollen. It is a suppurative process, involving the hair follicles and the sebaceous glands belonging to them. Small pustules form at the roots of the cilia, these break down, becoming ulcers, which scab over and cicatrise. The hair follicles are destroyed by the suppuration, and therefore the lashes do not grow again.

The clinical picture presents the lids red and swollen, a pustule here, an ulcer there, a scab or a cicatrix deficient in cilia, the remaining eyelashes often matted together by the dried secretion. The condition is an eczema of the margin of the eyelid. It is a chronic disease, which often extends over many years, and is the more serious of the two forms. Permanent damage may remain.

*Sequelæ.*—1. Destruction of the eyelashes, by suppuration of the hair follicles.

2. Trichiasis—cicatrisation produces a misdirection of the neighbouring cilia, so that they turn backwards towards the cornea. This may produce much irritation along with a superficial keratitis.

3. Hypertrophy of the lid border, due to the continued congestion and inflammatory swelling. This condition is spoken of as "tylosis," and affects mainly the upper lid.

4. Chronic conjunctivitis—an extension of the chronic inflammatory process from the lid margin to the conjunctiva.

5. Ectropion of lower lid, produced by cicatricial contraction. This involves the punctum lacrimale, which becomes everted, so that epiphora develops The consequent continual wetting of the skin of the lower lid results in excoriation and eczema. In turn this increases the ectropion and so the epiphora.

*Treatment.*—The condition is generally found in strumous children, often associated with phlyctenular ophthalmia. The general condition and the hygienic surroundings of the patients must be improved. Syrup ferri phos. co. or syrup ferri. iodid. in doses of a drachm are useful. Often an error of refraction is present; this should be looked for and corrected if necessary.

The local treatment should be non-irritating. The scales and scabs are first to be softened by the application of vaseline or olive oil, and then completely soaked off with an alkaline lotion, such as 15 grains of sodium bicarbonate to an ounce of water. The pustules must be opened every day and the involved cilia removed with epilation forceps. The healing of the ulcers is promoted by occasionally touching them with the nitrate of silver stick. The eyes should be bathed with the alkaline lotion three times a day, then dried, and an ointment of 8 grains of hydrarg. ammoniat. to 1 ounce of soft paraffin or benzoated lard applied. The white precipitate added to an ounce of ung. zinc oxide makes a useful ointment.

In more obstinate cases protargol massage before the application of the ointment is beneficial. It consists of a thorough rubbing into the hair follicles with a fine camel's hair brush of a 15 or 20 per cent. solution of protargol. A more recent treatment is the injection of anti-staphylococcic vaccine. In the pustules staphylococci, more frequently aureus, are found. The opsonic index of the patient to the organism found in the pustule is then ascertained. If this is low the injection of the corresponding anti-vaccine has been found, in some cases, of considerable value. A half c.c. is first injected subcutaneously, and after intervals of ten days the dose is increased up to 2 c.c. according to the results obtained.

